# Distribution of Tetrodotoxin in Pacific Oysters (*Crassostrea gigas*)

**DOI:** 10.3390/md19020084

**Published:** 2021-02-02

**Authors:** Monika Dhanji-Rapkova, Andrew D. Turner, Craig Baker-Austin, Jim F. Huggett, Jennifer M. Ritchie

**Affiliations:** 1 Centre for Environment, Fisheries and Aquaculture Science (Cefas), Barrack Road, Weymouth DT4 8UB, UK; andrew.turner@cefas.co.uk (A.D.T.); craig.baker-austin@cefas.co.uk (C.B.-A.); 2 Faculty of Health and Medical Sciences, University of Surrey, Guildford GU2 7XH, UK; j.huggett@surrey.ac.uk; 3 National Measurement Laboratory, LGC, Queens Rd, Teddington TW11 0LY, UK

**Keywords:** tetrodotoxin, Pacific oyster, *Crassostrea gigas*, organs, distribution, digestive gland

## Abstract

A potent and heat-stable tetrodotoxin (TTX) has been found to accumulate in various marine bivalve species, including Pacific oysters (*Crassostrea gigas*), raising a food safety concern. While several studies on geographical occurrence of TTX have been conducted, there is a lack of knowledge about the distribution of the toxin within and between bivalves. We, therefore, measured TTX in the whole flesh, mantle, gills, labial palps, digestive gland, adductor muscle and intravalvular fluid of *C. gigas* using liquid chromatography-tandem mass spectrometry. Weekly monitoring during summer months revealed the highest TTX concentrations in the digestive gland (up to 242 µg/kg), significantly higher than in other oyster tissues. Intra-population variability of TTX, measured in the whole flesh of each of twenty animals, reached 46% and 32% in the two separate batches, respectively. In addition, an inter-population study was conducted to compare TTX levels at four locations within the oyster production area. TTX concentrations in the whole flesh varied significantly between some of these locations, which was unexplained by the differences in weight of flesh. This is the first study examining TTX distribution in *C. gigas* and the first confirmation of the preferential accumulation of TTX in oyster digestive gland.

## 1. Introduction

Tetrodotoxin (TTX) is a thermostable and highly potent neurotoxin, most often associated with food poisoning due to the consumption of pufferfish (*Tetraodontidae* family) (reviewed in [1,2]). Besides pufferfish, which have been responsible for nearly 60% of TTX poisoning cases, other sources include edible marine gastropods (predominantly from family *Nassaridae*) and arthropods (*Carcinoscorpius rotundicauda*), contributing to 21% and 16% cases, respectively [3]. TTX has also been found in other marine animals, many of which are unlikely to pose a food safety risk, including starfish, marine worms from several phyla (Platyhelminthes, Nemertea, Chaeognata and Annelida), or terrestrial amphibians (reviewed in [1,2,4]).

Historically, TTX has been viewed as a food safety threat mainly in south-east and east Asia. Indeed, the first report of TTX in bivalve shellfish was from Japan in 1993, when the accumulation of marine biotoxins in scallops (*Mizuhopecten yessoensis*, formerly *Patinopecten*) was investigated [5]. However, no further studies on TTX occurrence in bivalves were reported until 2009, when TTX was found in New Zealand [6]. Despite a year-long study, which was prompted by cases of dog fatalities caused by TTX in sea slugs, and which involved 53 different marine vectors, endemic clam “Pipi” (*Paphies australis*) was the only species to contain TTX at multiple sites and different times of the year [6,7]. Further research on TTX in Japanese scallops and in *P. australis* from New Zealand has been conducted recently [8,9,10,11]. Elsewhere, TTX levels below 16 µg/kg were detected in several bivalve samples in China, collected during a market survey [12].

In Europe, the first investigation into TTX in various non-pufferfish vectors, including bivalves, was triggered by a non-fatal human poisoning case caused by ingestion of a trumpet sea snail (*Charonia lampas*), caught in Portuguese waters in 2007 [13,14]. The chemical analysis confirmed high levels of TTX (315 mg/kg) in the digestive gland of the animal involved in the poisoning episode [14], which was >10× higher than regulatory limit for TTX in Japan (2 mg/kg). The doses causing acute poisoning and lethality in humans, based on a series of intoxication cases, are not conclusive, with reported values between 4 and 42 µg/kg body weight [15]. After the first TTX intoxication case in Europe, the follow-up monthly screening study of 14 benthic species, including seven mussel samples (*Mytilus galloprovincialis*), confirmed TTX only in three gastropod species [16]. The first report on TTX in European bivalves found TTX (up to 120 µg/kg) in mussels (*Mytilus* spp.) and Pacific oysters *(Crassostrea gigas)* from southern England in 2013 and 2014 [17]. Soon after, TTX levels up to 203 µg/kg were documented from Greece in both whole flesh and digestive gland of multiple mussel samples (*Mytilus galloprovincialis*) and a venus clam (*Venus verrucosa*) sample, collected between 2006 and 2012, which would mark the earliest confirmed presence of TTX in European bivalves, dating back to 2006 [18]. The first findings of TTX in European shellfish prompted largescale (>1000 samples) screening studies of different bivalve species collected between 2014 and 2016 in the United Kingdom (UK) and between 2015 and 2017 in the Netherlands, where TTX concentrations up to 253 µg/kg were documented [19,20]. Other screening studies (<1000 bivalve samples) in France, Spain and Portugal did not find TTX, except five samples with levels < 12 µg/kg. To date, the highest TTX concentrations in European bivalves were reported in *M. galloprovincialis* (541 µg/kg), collected during a targeted study in north-east Italy [21]. TTX was also found in Sicily [22], albeit at low concentrations (<7 µg/kg).

The first TTX surveys in European bivalves have indicated a low prevalence given the size of the geographical area that has been examined, and the time period covered (reviewed by [23,24]). Consequently, the risk to a shellfish consumer for potential exposure to TTX appears to be small, supported by the absence of any confirmed TTX poisoning cases caused by the consumption of bivalves, either in Europe or worldwide. Nevertheless, there has been an increased interest in uncovering the origin of TTX, to identify the driving factors for its accumulation in bivalves and its temporal and depuration dynamics, in order to assess the risk to the shellfish consumer as well as to the shellfish industry. These efforts have been supported by the European Food Safety Authority (EFSA), who published a scientific opinion on TTX as an emerging food safety threat in Europe [15]. While considering an adult body weight of 70 kg, a large portion size of bivalves, and a proposed acute reference dose of 0.25 µg TTX eq./kg body weight, EFSA recommended the maximum permitted level (MPL) of 44 µg TTX eq./kg of bivalve. TTX at MPL and below is considered not to cause adverse effects in humans and was adopted in the Netherlands, the only country to date to formally include a TTX MPL for bivalves in the national biotoxin monitoring programme [20].

Knowledge of TTX distribution in bivalve populations, individual animals as well as organs, remains scarce. Biessy et al. (2018) examined TTX in different parts of clam *P. australis* and found the mean TTX concentrations in the siphons were significantly higher compared to any other dissected part (>400 µg TTX/kg versus <50 µg/kg in other organs) [9]. In contrast, sustained TTX levels (31–133 µg/kg) were found recently in the digestive gland of Japanese scallop over an eight-month period, while TTX was not detected in the remaining tissues [10]. Considering differences in ecology and physiology among bivalves, it is important to evaluate TTX distribution at organ level for individual species from different regions. To date, no information is available on TTX distribution within *C. gigas* tissues, which were previously reported to accumulate TTX in Europe [17,19,20], nor between different animals within any given population. In the UK, due to the declining trend of native flat oyster (*Ostrea edulis*) [25], the importance of *C. gigas* for shellfish industry has grown, and the annual production nearly doubled from 1,200 tonnes in 2013 to 2200 tonnes in 2018, the fourth largest production in Europe [26]. Annual oyster yield in Europe is estimated at 150,000 tonnes and the first sale value at EUR 550 million [27]. Therefore, investigating TTX occurrences and distribution in this commercially important species is of interest.

In this study, firstly we assessed the TTX dynamics in several *C. gigas* organs, intravalvular fluid as well as in the whole tissue during a TTX event in the summer of 2019. The proportions of TTX levels were compared between organ tissues over the period of two months. Secondly, TTX was measured in the whole flesh of 20 individual animals on the two separate occasions to assess intra-population variability. Thirdly, the distribution of TTX in several populations within one production area was also examined.

## 2. Results

### 2.1. TTX Recovery in C. gigas Matrices and Matrix Effect on TTX Signal

While extraction approaches were previously optimised for shellfish flesh [28], this has not been completed for intravalvular fluid samples. Therefore, six different treatments were applied to TTX-spiked fluid, as detailed in Section 4.3.2, and recoveries were compared (Table 1). The highest TTX recovery (91%) was measured when intravalvular fluid was neither acidified nor boiled, and, as such, forwarded for Solid Phase Extraction (SPE) clean-up without prior treatment. This approach, which was also the most time-efficient, was adopted for the time series analysis of intravalvular samples.

TTX recovery was assessed in each tissue, as detailed in Section 4.3.2. The highest levels of recovery were in the mantle and gills (86% and 81%, respectively), followed by adductor and digestive gland (68% and 60%, respectively). Only 37% of spiked TTX could be recovered from the labial palp homogenate (Table 2). The higher TTX losses in palps could be due to the addition of dichloromethane into the acidic extract, enabling SPE clean-up. Recovery in the two whole-flesh matrices was 66% and 76%, respectively, comparable to those reported previously for *C. gigas* whole flesh (80% ± 22%), spiked with similar TTX concentration (20 µg/kg) and processed following the same extraction and clean-up protocols [28].

Linear regression correlation coefficients were >0.99, confirming linear detection based on two bracketed TTX calibration curves, each comprised of six concentration levels, and prepared either in the solvent or in the matrix. Comparing linear regression slope gradients of both matrix-matched and solvent-based calibration solutions analysed alongside each other, minimal matrix effects were identified for intravalvular fluid and mantle matrices, with slope ratios at 100% and 104%, respectively. Gills, adductor muscle and whole flesh had moderate matrix effects (either suppression or enhancement), no greater than 34%. Digestive gland and labial palp tissues caused the biggest suppression (by 45%) of TTX signal (Table 2). The use of matrix-matched standards was, therefore, beneficial for the majority of matrices, in order to compensate for varying degrees of matrix influence on TTX signal.

### 2.2. Time Series Study: TTX Concentrations in C. gigas Tissues

**Figure 1:**
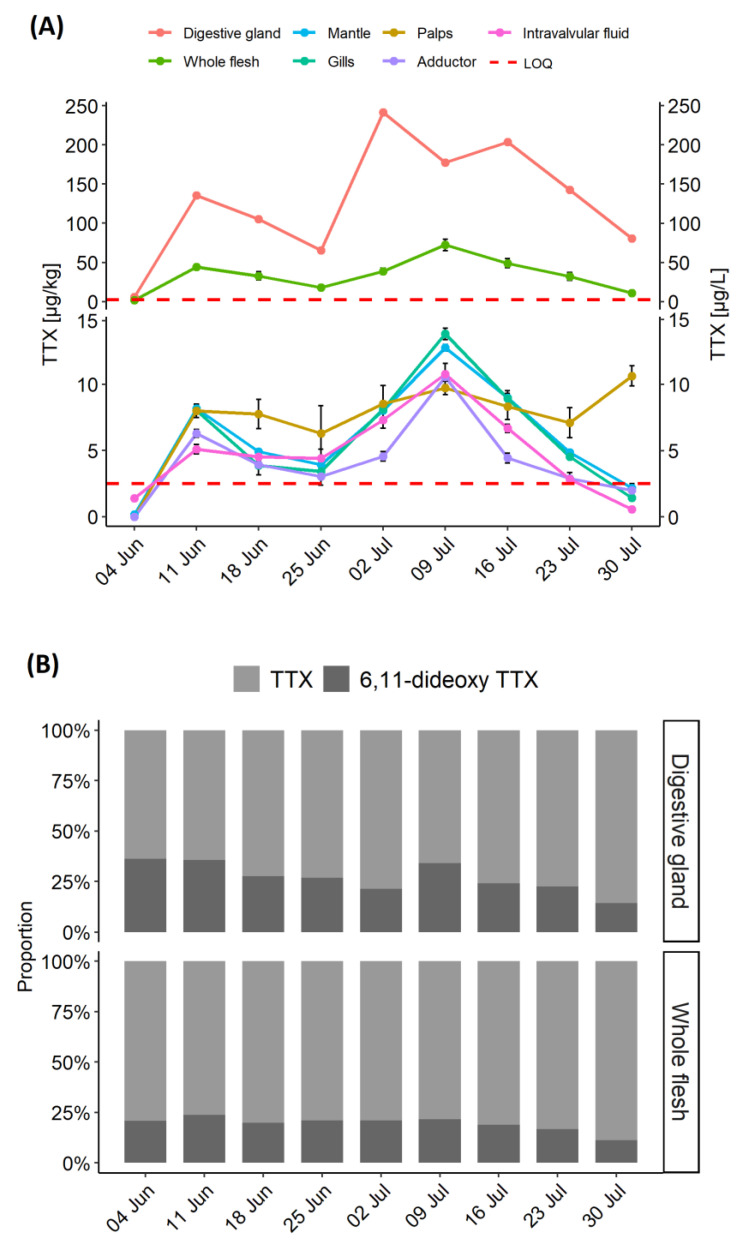
Time series of TTX showing (**A**) TTX concentration in various *C. gigas* matrices; (**B**) relative proportions of 6,11-dideoxy TTX and TTX in the *C. gigas* digestive gland and the whole flesh. The samples were collected between 4 June and 30 July 2019, TTX concentrations were expressed either in µg/kg (tissues) or µg/L (right-*y*-axis, intravalvular fluid only). Red dashed line represents an average Limit of Quantitation for various matrices (LOQ; 2.5 µg/kg).

*C. gigas* were analysed weekly between 7th May and 30th July 2019 from one sampling location with a history of TTX occurrence. TTX was detected for the first time on 29 May 2019 at levels below Limit of Quantitation (LOQ) in the whole flesh and the digestive gland (data not shown). TTX in the digestive gland increased more than 20-fold between 4 and 11 June (from 6.1 to 136 µg/kg), which was reflected in elevated TTX concentration in the whole flesh (Figure 1A). During the following two weeks, the levels in the digestive gland dropped by a half, before a sharp (~3.5 fold) increase to 242 µg/kg on 2 July 2019, which marked the peak of TTX concentration in the digestive gland. During the first half of July the levels remained high, followed by a rapid decrease (by 60%) in the latter two weeks of the month. TTXs continued to be analysed in the whole flesh beyond 30 July 2019 and the results confirmed the declining trend for TTX concentrations (data not shown).

The highest TTX concentrations were in the digestive gland; however, TTX was quantified in other tissues, albeit at low levels, between 11 June and 30 July 2019. In mantle, gills and adductor muscle, the highest TTX concentrations were observed on 9 July (12.8, 13.8 and 10.6 µg/kg, respectively), one week after the peak in the digestive gland. While the patterns of TTX dynamics, as well as weekly concentrations, were similar in mantle, gills and intravalvular fluid, levels in the adductor muscle were generally lower (Figure 1A), suggesting either slower uptake or faster depuration. The labial palps were the only compartment to show very little fluctuation in TTX concentrations, with an overall mean of 8.3 ± 1.4 µg/kg, and 17% variability, expressed as relative standard deviation (RSD), over eight sampling points.

In addition to TTX, 6,11-dideoxy TTX was quantified in the whole flesh and the digestive gland (Figure 1B). The average ratio between 6,11-dideoxy TTX and TTX in the digestive gland varied over the time, with the highest values, 57%, 55% and 52%, on 4 June, 11 June and 9 July, respectively. This coincided with period of sudden increase of TTX levels (Figure 1A). The average 6,11-dideoxy TTX proportions over the studied period were higher in the digestive gland than in the whole flesh (38% ± 14% and 24% ± 6%, respectively). Other TTX analogues (4,9-anhydro TTX, 11-deoxy TTX and 5,6,11-trideoxy TTX) were present in small quantities in the samples with the highest TTX concentrations.

**Figure 2:**
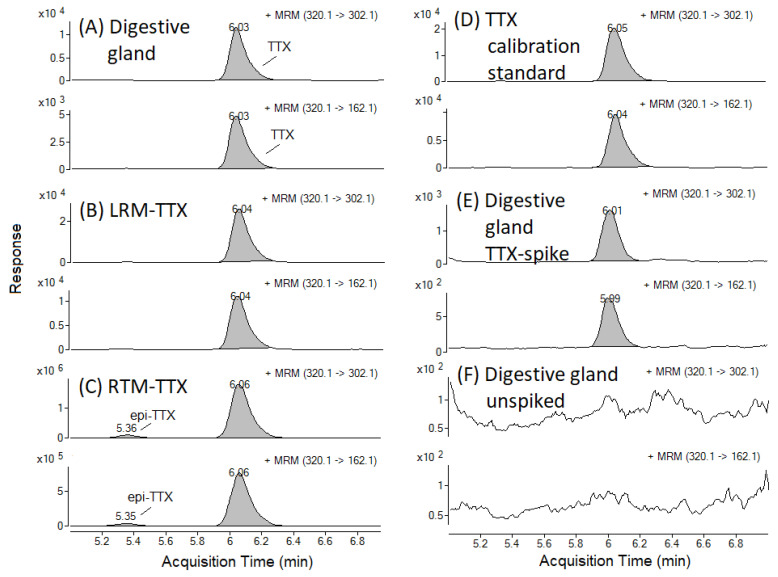
TTX Multiple Reaction Monitoring (MRM) chromatograms in the *C. gigas* digestive gland field sample collected on 9 July 2019 (**A**) and in the positive control samples: (**B**) Laboratory Reference Material (LRM-TTX-PO); (**C**) Retention Time Marker (RTM-TTX); (**D**) TTX calibration standard (5.18 ng/mL) prepared in the digestive gland matrix; (**E**) Digestive gland matrix collected in May 2019 and spiked with TTX. (**F**) shows TTX MRM in digestive gland matrix used for the recovery study and the preparation of matrix-matched standards.

An example of a TTX Multiple Reaction Monitoring (MRM) chromatogram from TTX-positive field sample is presented in Figure 2A, together with a selection of positive control samples (Figure 2B–E) and a digestive gland sample, which was absent of any detectable TTX. The latter was used for the recovery study and the preparation of matrix-matched standards (Figure 2F). The mean repeatability of triplicate analyses ranged between 1.9% for the mantle tissue to 14% in the palp tissue, with an overall mean value across seven matrices of 7.6%. TTX concentrations in positive control samples (LRM-TTX-PO and RM-TTX; Section 4.1) were within 30% of the expected value, as characterised previously [28,29]. TTX was absent in negative control (procedural blank) samples and in the matrices used for spiking experiments.

### 2.3. TTX Distribution in C. gigas Tissues

**Figure 3:**
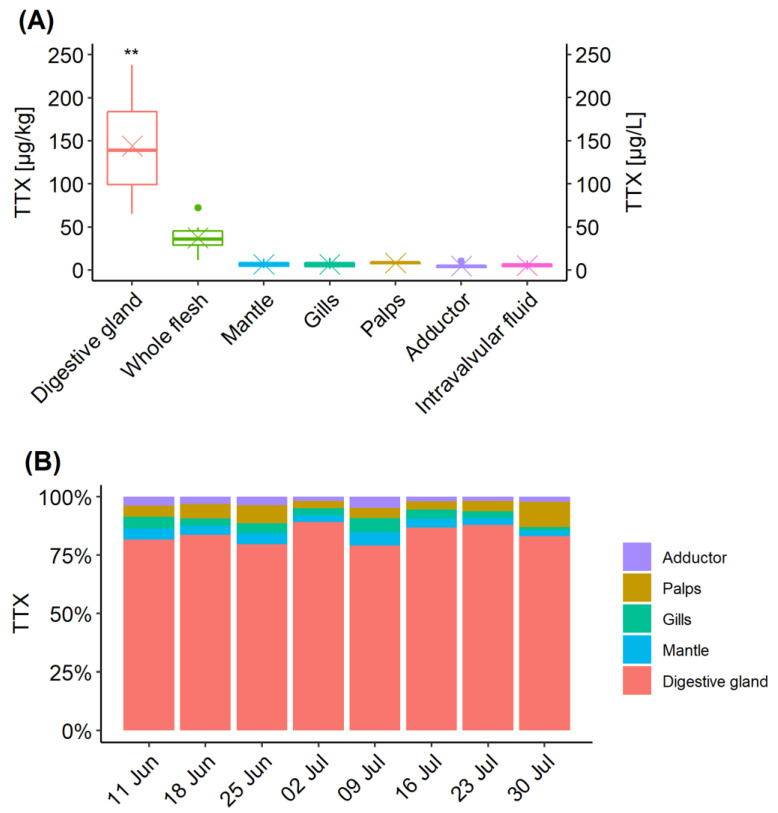
TTX distribution in *C. gigas* tissues collected between 11 June and 30 July 2019. (**A**) Box and whisker plot showing TTX concentration means (cross), median (horizontal line), 1 and 3 quartiles and outliers (dots) in each *C. gigas* tissue. ** means significance *p* < 0.01 when compared to other organs and *p* < 0.05 when compared to “Whole flesh”; (**B**) Relative proportions of TTX concentrations in individual *C. gigas* tissues over time.

The distribution of TTX in different organs was examined for each sampling occasion between 11 June and 30 July. Data from the 4 June were excluded, as only low levels were detected in the digestive gland (6.1 µg/kg) on that occasion. TTX concentrations in the digestive gland were significantly higher than in other organ tissues (*p* < 0.01) and the whole flesh (*p* < 0.05) (Figure 3A). Normality was confirmed by Shapiro-Wilk’s test of normality, followed by Welch paired *t*-test with “Bonferroni” adjustment of *p*-value. Changes in proportions of TTX concentrations over time are illustrated in Figure 3B. The highest proportion of TTX concentration in the digestive tissue was on 2 July (89%), coinciding with the highest mean TTX concentration in this organ (Figure 1A), while the lowest (79%) was only one week later, due to elevated contributions from other organs, mainly from gills, mantle and adductor muscle. TTX proportions in labial palps varied between 3% (2 July) and 11% (30 July), despite showing very little fluctuations in TTX concentrations (Figure 1A). The average proportion of TTX concentration in the digestive tissue was 84% ± 3.8%. The proportions in all other tissues were, on average, between 3 and 6% of TTX, although overall variability (RSD) was high, between 30 and 46%.

**Figure 4:**
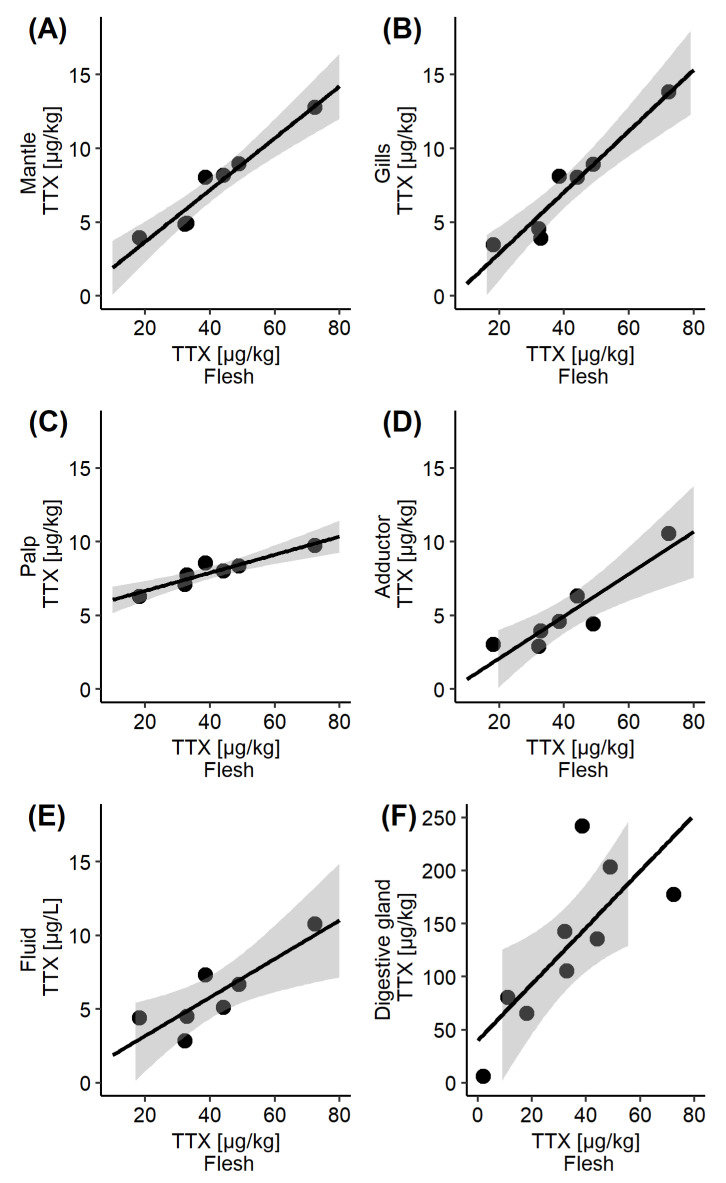
Scatter plot of the mean TTX concentrations in the whole flesh, collected between 11 June and 23 July 2019, and the corresponding mean TTX concentrations in various *C. gigas* matrices: (**A**) Mantle; (**B**) Gills; (**C**) Palps; (**D**) Adductor; (**E**) Intravalvular fluid; (**F**) Digestive gland, which also included datapoints from 4 June and 30 July. The fitted line represents linear regression, together with the 95% confidence interval.

TTX concentrations in the whole flesh reflected the TTX dynamics in some other organs (Figure 1A) and the visual indication was further explored by linear regression analysis (Figure 4). A significant linear correlation has been confirmed between the whole flesh and the mantle (r^2^ = 0.937, *p* < 0.001), gills (r^2^ = 0.922, *p* < 0.001), palps (r^2^ = 0.883, *p* < 0.01). adductor (r^2^ = 0.831, *p* < 0.01) and intravalvular fluid (r^2^ = 0.727, *p* < 0.05). More moderate linear correlation was observed between TTX concentrations in the whole flesh and the digestive gland (r^2^ = 0.596, *p* < 0.05), which was likely to be caused by a one-week time lag between TTX reached its peak in each of these tissues (2 July and 9 July, respectively, Figure 1A).

### 2.4. Intra-Population TTX Variability

**Figure 5:**
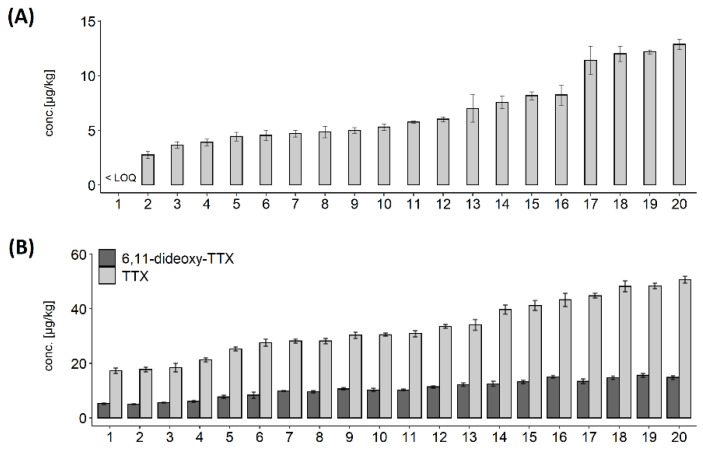
Variability of TTX toxins in individual *C. gigas*. (**A**) TTX concentrations in twenty individual *C. gigas* animals from Batch 1, arranged in ascending order. Animal 1 contained TTX below Limit of Quantitation for the whole flesh (LOQ; 0.8 µg/kg); (**B**) TTX and 6,11-dideoxy-TTX concentrations in twenty individual *C. gigas* animals from Batch 2, arranged in ascending order.

**Figure 6:**
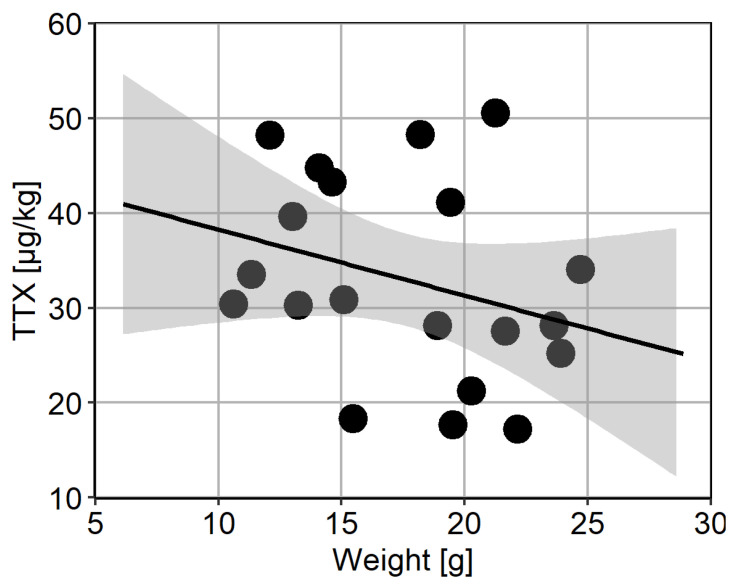
Scatter plot of the mean weight of flesh and the corresponding mean TTX concentrations from individual animals. The fitted line represents linear regression (R^2^ = 0.0964), together with the 95% confidence interval.

Two separate batches of 20 animals were used to assess intra-population variability. TTX was present in all animals from Batch 1; however, one animal contained levels below LOQ. The concentrations in other 19 animals ranged between 2.7 and 12.9 µg/kg, with median 5.7 µg/kg, mean ± s.d. 6.9 ± 3.2 µg/kg, and overall variability (RSD) was 46% (Figure 5A). The mean repeatability of triplicate analyses in Batch 1 was 8%. TTX concentrations in animals from Batch 2 was higher than in Batch 1 and ranged between 17.3 and 50.6 µg/kg, with median 30.7 µg/kg, mean ± s.d. 32.9 ± 10.6 µg/kg, and overall variability (RSD) was 32% (Figure 5B). In addition, the TTX analogue 6,11-dideoxy TTX was quantified above LOQ in all individuals from Batch 2, ranging from 5.1 to 15.6 µg/kg, with average 10.5 µg/kg and overall variability 32% (Figure 5B). The ratio between 6,11-dideoxy TTX and TTX was stable at 32 ± 2%. The mean repeatability of triplicate analyses for TTX and 6,11-dideoxy TTX was 4% and 5%, respectively. Finally, no correlation between TTX concentration and weight of the flesh from individual animals was found over the concentration range tested (Figure 6).

### 2.5. Inter-Population TTX Variability

**Figure 7:**
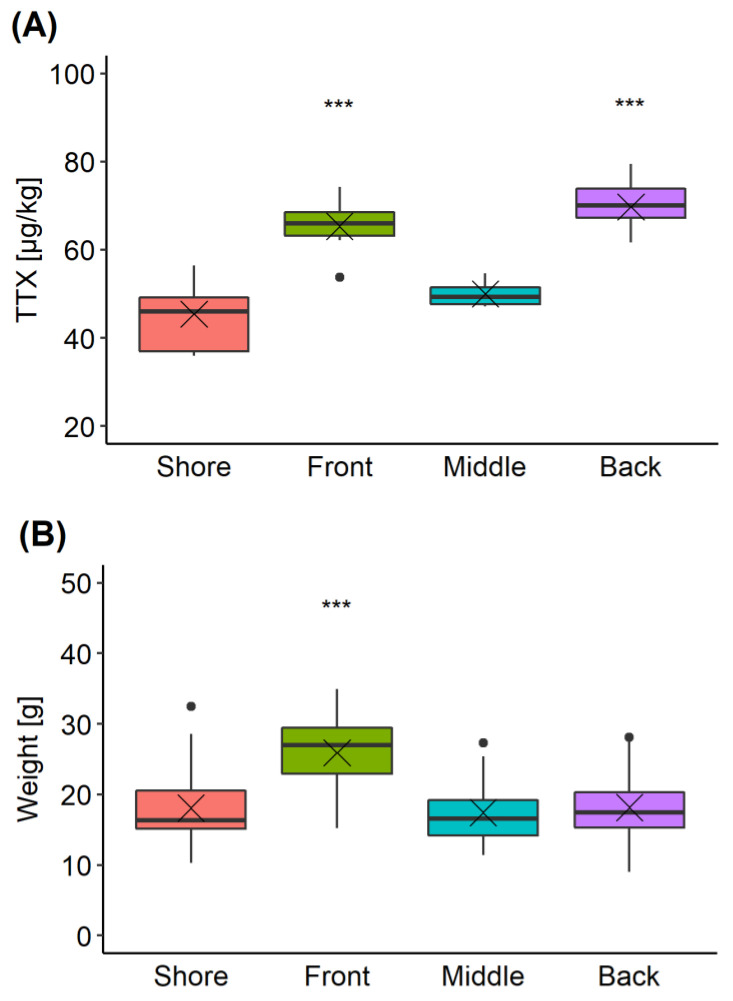
Box and whisker plot showing means (cross), median (horizontal line), 1st and 3rd quartiles, outliers (dots) for TTX (**A**) and weight of animals (**B**) in each of four locations (“Shore”, “Front”, “Middle” and “Back”) within the oyster production area. *** means significance *p* < 0.001 (pairwise *t*-test) when compared to “Shore” and “Middle” (**A**), or when compared to other locations (**B**).

*C. gigas* production sites can extend across large areas. To explore the variability in TTX concentrations present in oysters located at different parts of a single production area, samples were collected from four sites during a single harvesting trip (Section 4.3.4). The overall mean TTX concentration in all oysters was 58 ± 8 µg/kg, with an overall variability (RSD) of 20%. TTX concentrations in oysters obtained from “Front” and “Back” locations were 65 ± 4 and 70 ± 5 µg/kg, respectively, significantly higher than those found in “Shore” (45 ± 8 µg/kg) and “Middle” (50 ± 2 µg/kg) locations (pairwise *t*-test with “Bonferroni” adjustment of *p*-value) (Figure 7A). The variability associated with the TTX mean values, was between 4% and 8%, except for the “Shore” location, where it reached 17% and it was largely due to one batch returning a lower TTX result (37 ± 0.6 µg/kg) compared to other two batches within the same location. The mean repeatability of replicate analyses for each batch of ten oysters was 6%.

**Figure 8:**
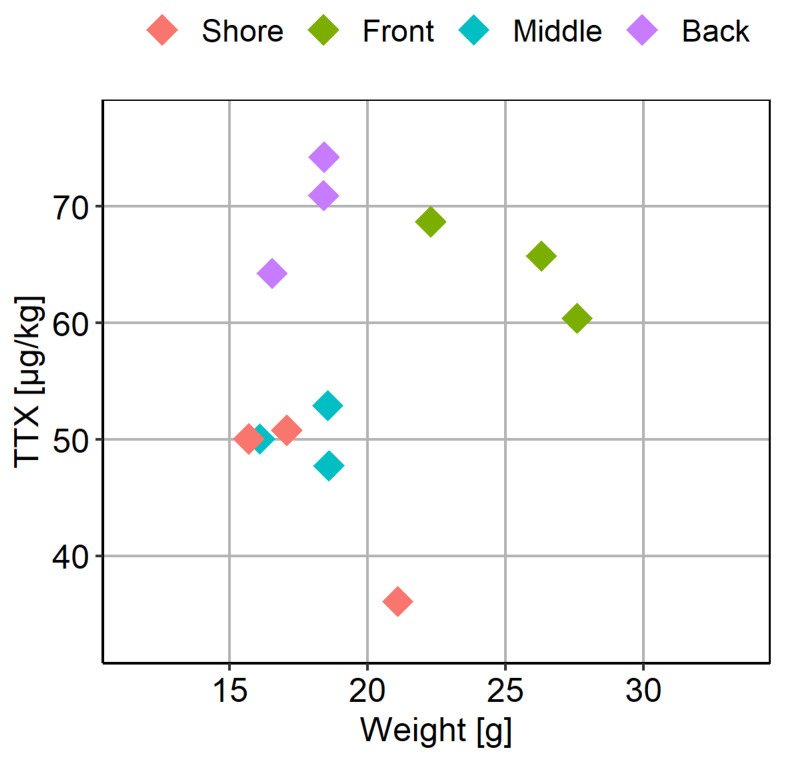
Scatter plot of the mean weight of flesh and the corresponding mean TTX concentrations in batches of *C. gigas*, each represented by ten individual animals, and collected from four locations (“Shore”, “Front”, “Middle” and “Back”) within the oyster production area.

The average weight of the *C. gigas* flesh was 20 ± 6 g; however, the mean weight in the “Front” location was significantly higher compared to the other locations (Figure 7B). Considering both TTX and weight values were higher in the “Front” location, the relationship between these two variables was further examined (Figure 8). Overall, however, no linear correlation between TTX concentration and weight of the flesh was found (R^2^ = 0.0846, *p* = 0.359).

## 3. Discussion

The first TTX screening of bivalve shellfish in the United Kingdom identified a limited number of locations with TTX occurrence in early summer [19]. The main aim of our study was to examine TTX distribution in shellfish from one of the sites with a history of TTX occurrence, and with TTX concentrations high enough to enable comparative analyses. Although we intended to include multiple bivalve species, the local restrictions meant that *C. gigas*, which has been grown commercially in the area, was the only available species for our study. When comparing TTX concentrations in different *C. gigas* tissues, significantly higher levels were found in the digestive gland from the onset of TTX accumulation at the beginning of June until the end of July 2019, when the TTX concentrations decreased. No further increases in TTX concentrations were observed in oysters during the following weeks and months of 2019, as confirmed by the long-term analysis of the whole tissue (data not shown). High proportion of TTX found in the digestive gland of *C. gigas* is in contrast to the low proportion found in the digestive gland of *P. australis* from New Zealand. This clam, which has been the only bivalve species subjected to TTX distribution studies to date, accumulated significantly higher TTX levels in siphons compared to other organs [9]. In the follow up experiment, when *P. australis* was kept in laboratory conditions and fed a TTX-free diet, the digestive gland was the only organ from which TTX depurated quickly (within 21 days) and completely while it remained present in other organs (siphons and the foot) for the duration of the study (150 days). The overall TTX concentrations in the whole flesh decreased significantly (by 60%) after 150 days, suggesting the clam sources TTX exogenously from the food [8].

Both *P. australis* and *C.gigas* are suspension-feeding lamellibranchiate bivalves, capturing the particles suspended in water by ctenidium, which also serves as the respiratory organ (gill). Burrowing species, including *P. australis*, utilise a pair of siphons to inhale and exhale water towards and from the gills. *C. gigas* do not bury and have no siphon; the water exchanges through an aperture instead (reviewed by [30]). Therefore, when comparing TTX distribution in organs of bivalve species, the differences in their anatomy, physiology and ecology should be considered. *P. australis* was identified as a slow detoxifier for TTX by Biessy et al. (2019) [8], who also hypothesised TTX could be preferentially stored in siphons using unique TTX-binding proteins similar to those found in the puffer fish *Takifugu pardalis* [31] or crab *Hemigrapsus sanguineus* [32]. An ecological role of TTX for *P. australis* has been proposed, such as acting as a deterrent to siphon-nipping predators when clams bury in the sand but leave their siphons above the sediment to filter feed [9,33]. In our study, the TTX event lasted only approximately two months, aligning with earlier reports on the quick accumulation and depuration of TTX for mussels (*Mytilus* sp.), *C. gigas*, and hard clams (*Mercenaria mercenaria*) in their native environments in the UK and the Netherlands [19,20]. Consequently, it is unlikely that TTX is being stored purposely in these species in order to fulfil an ecological role. Interestingly, Japanese scallops *(M. yessoensis)* sustained TTX levels up to 133 µg/kg in their digestive glands for eight months. TTX was not measured in other organs separately; however, the remaining flesh, left over after the dissection of the digestive gland, did not contain TTX [10]. The mechanism of TTX retention in digestive glands and whether it might have any ecological role in scallops remains unclear. It should be noted that scallops are capable of retaining other water-soluble toxins like domoic acid for several months [34,35,36], while other species detoxify it very quickly [36,37]. Another water-soluble toxin, saxitoxin (STX), which has a different chemical structure but almost identical toxicological effect as TTX, was found to be retained by clam species *Spisula solidissima* and *Saxidomus gigantea* for one and two years, respectively [38,39], while it was depurated from the other bivalve species within weeks [40]. For any future investigations and discussions about TTX distribution in bivalves, interspecies differences should be considered.

In our study, the highest concentrations of TTX were found in the digestive gland, significantly higher than in any other organ at any point during the monitored period. It can, therefore, be hypothesised that *C. gigas* is likely to accumulate TTX from a possible food source. Filter-feeding bivalves can efficiently retain cells > 7 µm from water by ctenidia; however, some species can utilise particles > 1 µm [41]. As such, a wide range of organisms could be available as a source of nutrients, with microplankton and nanoplankton being the most commonly recognised. The hypothesis of phytoplankton being a source of TTX in bivalves was articulated for the first time by Kodama et al. (1993) [5], when TTX occurrence in scallops coincided with an *Alexandrium tamarense* bloom. The authors later detected TTX and STX in *A. tamarense* culture (OF84423D-3) [42], however more recent studies contradicted this observation. STX and TTX were both found in *M. yessoensis* in 2017, however TTX did not correlate with *A. tamarense* densities [10]. A co-occurrence of STX and TTX has also been documented in mussels from Sicily, which coincided with *Alexandrium minutum* and *Alexandrium pacificum* blooms. However, toxin profile analysis of concentrated water samples collected during the bloom did not reveal TTX [22]. A link between the TTX and the dinoflagellate *Prorocentrum cordatum* (formerly *P. minimum*) was suggested by Vlamis et al. (2015) [18]. The follow-up study did not confirm TTX, but revealed TTX-like compounds in two *P. cordatum* reference strains, showing the same mode of action as TTX in an electrophysiological assay [43]. The link between *P. cordatum* and TTX in bivalves was also investigated in England, however the time series of these two parameters did not suggest a correlative relationship [19]. It should be noted that *C. gigas*, as well as other bivalve species, exhibit selective grazing, based not only on size, quality and digestibility of the available cells, but also other factors like the quality of the seston [30]. As such, diversity of cells digested by bivalves might not always reflect the community of phytoplankton and other organisms in water column [44]. Even though the phytoplankton or other digested food has not been confirmed as TTX source for bivalves, its role in TTX accumulation in *C. gigas* cannot be excluded. In our intra-population study, TTX was detected in all 20 individuals (Figure 5), with average variabilities of 32% and 46%, for two respective batches collected on different days. The intra-population variability of TTX was within the range of variability of other water-soluble toxins, like STX and domoic acid [45,46], which are known to accumulate in bivalves through filter feeding on toxin-producing phytoplankton. However, other factors have been shown to influence and expand toxin variability within populations, including animal size, filtration rate, reproductive condition and water depth for subtidal populations [47,48,49,50,51]. The results from our inter-population study might also contribute to this discussion. Average TTX levels in oyster population were significantly higher in the “Front” and the “Back”, when compared to the “Middle” part of the main area and to the population from the “Shore”. Interestingly, the main growing area is influenced by strong tidal streams, exceeding 1 kts during both spring and neap tides. However, the velocity of the tidal streams at the “Shore” location is generally much lower (0.2–0.6 kts) [52]. The oyster production area is situated in the intertidal zone, with the majority of the oyster trestles exposed at low tide. As such, the distribution of phytoplankton and other sources of food for oysters is likely to be influenced by the tidal streams. Lower TTX levels in “Shore” oysters could have been a result of reduced exposure to TTX producer/bearer. The oyster population in the “Middle” of the main growing area might be sheltered from the tidal streams because of the trestles surrounding it. The shellfish grower reported that middle trestle rows contain stock and young animals; however, commercial-size animals were picked for the study. The flesh weight of individual batches from the “Middle” was not significantly different to the ones from the “Shore”, and the “Back”, hence the differences in weight/size of the animals could not explain the differences in mean TTX concentrations. Unless more factors are investigated, the reasons for significant differences in TTX levels between populations in this relatively small area cannot be elucidated.

The presence of TTX in evolutionary diverse organisms (reviewed in [1,2,4]) prompted speculation of an exogenic origin of the toxin, which is thought to be acquired by the animal either from the diet [53], or from associated bacteria [4,54]; however, the bacterial biosynthetic genes responsible for TTX production are yet to be described. Some earlier evidence on the TTX presence in bacterial cultures has come under scrutiny due to the limitations of unspecific fluorescence-based detection methods utilised at the time [55,56,57]. Thanks to a wider availability of highly specific and selective LC-MS-based chemical methods, more reports, confirming TTX in bacterial cultures isolated from various TTX-bearing organisms, have recently emerged [17,58,59,60,61]. The only TTX-producing bacterial genus, isolated and identified to date from bivalves (specifically from *Mytilus* spp. and *C. gigas*), was *Vibrio* [17]. However, the origin of TTX-producing *Vibrio* population remains unclear; whether it was transported into bivalves through filter-feeding, either as free-living cells or associated with some other organisms/detritus, or whether the *Vibrio* growth was promoted inside the bivalve. It is less probable that free-living bacteria smaller than 1 µm are actively retained by *C. gigas*, as the study by Palmer et al. (1980) [62] reported only 50% retention of 1.8 µm-sized particles by eastern oyster (*Crassostrea virginica*). *Vibrio* uptake from sea water as well as growth within oysters was also documented [63], however the preferential accumulation of *Vibrio* in oyster digestive gland in the native environment has not been unequivocally confirmed. Accumulation of TTX in *C. gigas* digestive gland revealed by this study could indicate introduction of TTX-producing bacteria facilitated by the digested organisms.

Despite sparse evidence on microplankton origin, and the widely preferred hypothesis of bacterial origin of TTX, there is a rationale behind the role of microplankton in TTX accumulation in bivalves. Bivalves as filter feeders can accumulate toxin compounds produced by phytoplankton themselves [64] or potentially by bacteria co-habiting or living in a symbiotic relationship with organisms digested by bivalves. Biotic interactions between bacteria and phytoplankton have been studied more intensively in recent years and are considered to be complex [65,66,67]. There are many factors yet to be considered; however, this paper will facilitate more targeted analyses in future TTX research of bivalves and specifically of Pacific oysters. The presented evidence on preferential accumulation of TTX in its digestive gland could form a basis for studying microplankton and microbial communities in water and the digestive gland before, during and after period of TTX accumulation.

## 4. Materials and Methods

### 4.1. Reagents and Chemicals

Reagents for the sample extraction and subsequent Solid Phase Extraction (SPE) sample clean-up were HPLC grade or equivalent. Reagents for mobile phases and instrument washes such as water, Acetonitrile (MeCN), formic acid and 25–31% ammonium hydroxide were all of LC-MS grade. TTX certified reference material (CRM) (Cifga, Lugo, Spain) and non-certified TTX material (Enzo Life Sciences, Exeter, UK) were purchased to prepare TTX stock solutions. One Cifga TTX CRM ampoule (containing 25.9 ± 1.3 µg/g TTX) was opened and the contents diluted ten-fold with 0.25% acetic acid in deionised water and stored at <−15 °C. Enzo TTX powder was solubilised in 0.25% acetic acid to achieve concentration 10 µg/mL and stored at <−15 °C. Both stock solutions were further diluted ten-fold to enable preparation of TTX calibration solutions over six concentration levels (0.04–64.75 and 0.05–100 ng/mL for Cifga and Enzo solutions, respectively). The diluent was either 80% MeCN with 0.25% acetic acid (solvent calibration solutions), or the shellfish tissue extract, which had been SPE-cleaned and diluted with MeCN in 1:3 ratio (matrix-matched calibration solutions). Once prepared, the calibration solutions were kept in the refrigerated autosampler (< 10 °C) and used within a week.

In the absence of certified TTX-positive control materials in shellfish matrix, the following TTX positive control samples have been utilised in the study:

1. Laboratory Reference Material (LRM-TTX-PO), containing TTX (181 µg/kg) and a range of TTX analogues, was prepared by Cefas as described by Turner et al. (2017) [28];
2. Non-certified mussel reference material (RM-TTX-Mus), containing TTX (2275 µg/kg of wet tissue homogenate) and a range of TTX analogues [29], was obtained from National Research Council Canada (NRCC, Halifax, Canada). After 0.35 ± 0.05 g of the freeze-dried powder was reconstituted in 1.65 mL of deionised water, vortex mixed for 30 s and sonicated for 1 min in ultrasonic bath, it was subjected to TTX extraction procedure. This material is not currently commercially available, and therefore it was extracted only once, and the cleaned extract was diluted weekly to check the performance of the instrumental detection method;
3. Retention time marker (RTM-TTX), used for qualitative identification of retention times of TTX and multiple TTX analogues, was prepared from freeze-dried tissue of the sea slug *Pleurobranchaea maculata* (Cawthron Natural Compounds, Nelson, New Zealand), as described by Turner et al. (2017) [28].

### 4.2. Toxin Analysis

#### 4.2.1. Extraction, Clean-Up and Dilution

Homogenised shellfish tissue was subjected to a single dispersive extraction using 1% acetic acid in 1:1 (*w/w*) ratio, as previously validated [28]. For the adductor muscle, 1:2 ratio was adopted to allow thorough homogenisation with the Bio Gen PRO 200 homogeniser (Pro Scientific, Oxford, MS, USA). The resultant mixture was vortex mixed for 5 min at 2500 rpm before being placed into a boiling water bath for 5 min. Samples were then cooled for 5 min in cold running water and further vortex mixed for 5 min (2500 rpm). Samples were centrifuged at 4500 rpm for 10 min at 20 °C, then 1 mL of supernatant was pipetted into a 5 mL polypropylene tube and forwarded to SPE. Intravalvular fluid was processed in six different ways to evaluate the effect of acidification and boiling on the TTX recovery (Table 1). No acid was added for A and B treatment, whereas for treatments C, D and E, F either glacial acetic acid (final pH = 3.75), or 1% acetic acid (*v/v* = 1:1; final pH = 3.22) were added into intravalvular fluid, respectively. Samples for each treatment were either boiled or this step was skipped. A negative control (1% acetic acid) and a positive control, either LRM-TTX-PO or TTX-spiked material, were processed with each batch of samples. For removal of some matrix interferences, mainly salts, a Gilson Aspec XL-4 SPE liquid handler, and Supelclean ENVI-Carb 250 mg/3 mL cartridges (Merck Life Science UK Limited, Gillingham, UK), were utilised. However, the labial palp extracts had to be purified prior to SPE clean-up by mixing the extract with dichloromethane at 1:1 (*v/v*) ratio, to allow the extract passing through SPE cartridges. Immediately after manual shaking and vortex mixing twice at 2500 rpm for 1.5 min, the mixture of dichloromethane and the extract was centrifuged at 4500 rpm for 10 min. The upper layer of clarified extract was pipetted out and subjected to manual SPE using vacuum manifold. For both, automated or manual SPE clean-up, the conditioning, loading and elution was performed as described by Turner et al. (2017) [28]. The eluent was vortex-mixed before 100 µL aliquots were diluted with 300 µL MeCN in 700 µL Verex polypropylene autosampler vials (Phenomenex, Manchester, UK).

#### 4.2.2. Liquid Chromatography—Tandem Mass Spectrometry

An Agilent (Manchester, UK) 1290 Ultra High-Performance Liquid Chromatography system coupled to an Agilent 6495B tandem quadrupole mass spectrometer (MS/MS) was used for TTX analysis. Since a Hydrophilic Interaction Liquid Chromatography (HILIC) column has been utilized (Acquity BEH Amide, 1.7 µm, 2.1 × 150 mm, in conjunction with a VanGuard BEH Amide guard cartridge, Waters), the analysis is referred to as HILIC-MS/MS. Mobile phase composition, mobile phase gradients, injection volume, autosampler and column temperature settings were as described in previous work [28,58], with slight modifications of the gradient to suit the Agilent instrument. MS/MS conditions and the electrospray ionisation (ESI) interface were as follows: gas temperature 150 °C, gas flow 15 L/min, nebulizer gas 50 psi, sheath gas temperature 400 °C, sheath gas flow 12 L/min, Capillary voltage 2500 V. Multiple Reaction Monitoring (MRM) were acquired in positive ionisation mode and were taken from Turner et al. (2017, 2018) [28,58]. Primary (quantitative) and secondary (qualitative) MRMs for each analogue were as follows: TTX and 4-epi-TTX (320.1 > 302.1, 162.1), 5,6,11-trideoxy TTX (272.1 > 254.1, 162.1); 11-nor TTX-6-ol (290.1 > 272.1, 162.1); 4,9-anhydro TTX (302.1 > 256.1, 162.1); 4,9-anhydro-5,6,11-trideoxy TTX (254.1 > 236.1, 162.1); 5-deoxy TTX (304.1 > 286.1, 176.1), 11-deoxy TTX (304.1 > 286.1, 176.1); 11-oxo TTX (336.1 > 318.1, 162.1); 4,9-anhydro-11-oxo-TTX (318.1 > 300.1, 162.1) and 6,11-dideoxy-TTX (288.1 > 270.1, 224.1). MRM transitions for arginine and hydroxy-arginine were also assessed, in order to evidence effective chromatographic separation of TTX from these matrix co-extractives, which are known to affect TTX quantitative recovery and, therefore, method accuracy [28].

The presence of the additional TTXs analogues was carried out by comparing both quantitative and qualitative MRM peaks and their associated ion ratios against those established in the concentrated calibration solution and the positive controls, specifically RTM-TTX. The Limit of Quantitation (LOQ) for *C. gigas* whole flesh was 0.8 µg/kg, and, on average, 2.5 µg/kg for organ matrices, based on values obtained from TTX-spiked samples.

### 4.3. C. gigas Collection and Preparation

#### 4.3.1. Time Series of Tetrodotoxins in *C. gigas*

*C. gigas* were harvested from a classified shellfish production area in southern England. The exact location is kept anonymous to protect the shellfish producer. Live animals were transported to the laboratory in a chilled cool box and kept at 10 ± 2 °C until processing (within 25 h after the collection).

**Figure 9:**
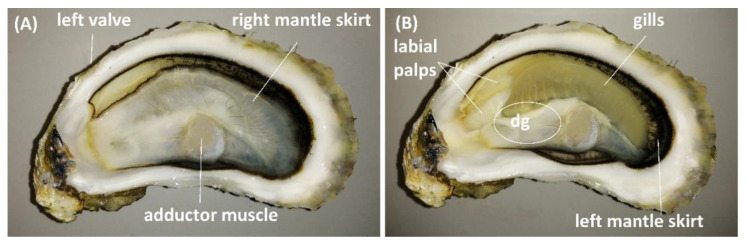
*C. gigas* tissues analysed for TTX. *C. gigas* with right valve removed (**A**), and right valve and right mantle skirt removed (**B**). The white circle indicates an approximate location of a digestive gland (dg) under the superficial tissues.

For the analysis of TTX over time, 25 animals were collected weekly between 7 May and 30 July 2019 and split into two batches. The whole flesh and intravalvular fluid from the first batch (12–13 animals) was pooled, respectively, to create representative samples for each sampling day. The second batch was forwarded for organ dissection, and a representative sample per organ type was created by pooling organ tissues from 11 to 12 animals. Shells were cleaned under running water and sprayed with 1% Virkon disinfectant. Animals were opened aseptically, the intravalvular fluid was drained into a sterile beaker, and the flesh was placed either in a sterile beaker (whole flesh) or on a sterile Petri dish (for organ dissection). After homogenisation (Waring Commercial, Stamford, CT, USA), three 5 g aliquots of flesh were weighed out in 50 mL polypropylene tubes and stored at −20 °C for subsequent TTX analysis. Animals from the second batch were aseptically dissected to obtain the anterior part of right mantle skirt (lining right valve), gills, labial palps, digestive gland and the striated “quick” part of the adductor muscle (Figure 9). The central part of the digestive gland, which included the stomach, was separated from the surrounding tissue. Vessels with shucked flesh and dissected tissues were kept cool (on ice or in the incubator set at 10 ± 2 °C) throughout the procedure. Samples were homogenised by Bio Gen PRO 200 homogeniser (Pro Scientific, Oxford, MS, USA) and stored at −20 °C until TTX analysis. One 2.5 ± 0.01 g aliquot per each organ tissue per sampling day was extracted, with the exception of three palp samples, where less than 2.5 g material was available (1.50, 1.45 and 2.01 g, respectively). Each organ tissue sample was analysed by HILIC-MS/MS in triplicates.

#### 4.3.2. TTX Recovery

Oyster tissues and intravalvular fluid, collected from the oyster farm in May 2019 or in March 2020, and absent of detectable TTX, were fortified with Enzo TTX stock solution (Section 4.1) at concentration 25 µg TTX/kg wet tissue or 50 ng/mL of intravalvular fluid, respectively. For each organ tissue, three 2.5 ± 0.01 g TTX-spiked samples and one 2.5 ± 0.01 g unspiked sample were subjected to the TTX extraction procedure. For the whole flesh, the aliquots were 5.0 ± 0.01 g. Each extract was analysed by HILIC-MS/MS in triplicate and the concentrations quantified using matrix-matched calibration solutions (Section 4.1). TTX recovery in intravalvular fluid (2 mL aliquots) was assessed after six different treatments (Table 1) to evaluate the most effective approach for the time series samples.

#### 4.3.3. Intra-Population Variability

To assess the intra-population variability of toxin concentrations in the whole flesh of individual *C. gigas*, 20 animals were collected on 27 June 2019 (Batch 1), and a further 20 on 9 July 2019 (Batch 2). After shells had been cleaned under running water, they were opened one by one and the whole flesh transferred into 50 mL polypropylene tubes. The weight of each animal was recorded, and the samples were stored at −20 °C until TTX analysis. Shellfish flesh from individual animals was thawed before it was homogenised with a T 25 digital ULTRA-TURRAX™ (IKA™ England Ltd., Oxford, UK) at 12,500 rpm for 30 s. One 5 g aliquot per animal was weighed out into 50 mL tubes and subjected to the TTX extraction procedure. All 20 samples in each batch were processed simultaneously and analysed by HILIC-MS/MS in triplicates in a single analytical run to minimise variability associated with the method.

#### 4.3.4. Inter-Population Variability

**Figure 10:**
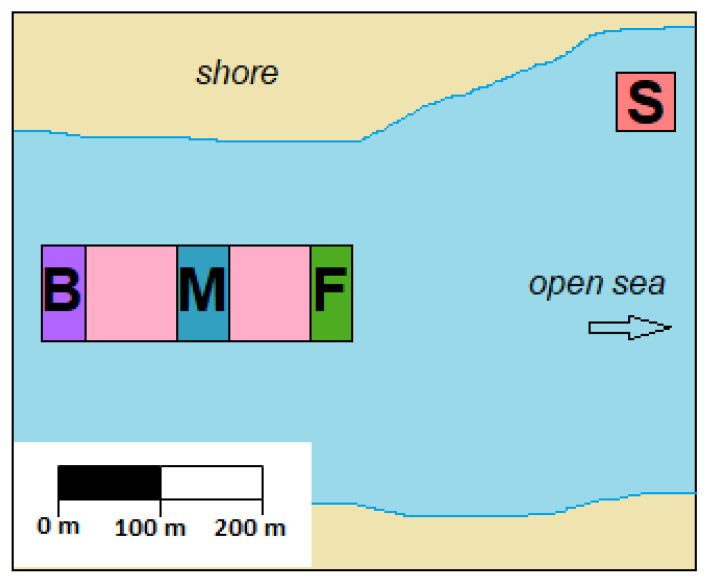
A simplified scheme of *C. gigas* farm, highlighting the locations sampled for inter-population variability: “S” = Shore; “F” = Front; “M” = Middle; “B” = Back.

To assess TTX variability between populations, 120 oysters were harvested during low tide on 29 June 2020 from four locations within one oyster production area. Three locations were within the main growing area, while the fourth one was approximately 200 m away from this area and was closest to the shore (Figure 10). Oysters had been refrigerated before they were transported to the laboratory, where they were stored at −20 °C until they were processed. The oysters were left to gradually defrost, the intravalvular fluid was drained and weight of flesh was recorded. Each sampling location was represented by 30 individual oysters, which were split evenly into three batches. Flesh from ten oysters in each batch was pooled and homogenised in a blender (Waring Commercial, Stamford, CT, USA). Three 5 g aliquots for each batch of oysters were weighed and stored at −20 °C. All 36 samples were processed for TTX simultaneously and analysed by HILIC-MS/MS in triplicates in one instrumental batch.

### 4.4. Data Analysis

TTX data were analysed using Agilent Technologies MassHunter Workstation software (version V.B.08.00). Because Enzo stock solution contained 81% ± 4% TTX in comparison to the Cifga TTX CRM [28], TTX in spiked samples was quantified using Enzo calibration solutions (Section 4.1) to compensate for the difference. TTX in other samples were quantified against Cifga calibration solutions. In order to evaluate and compensate for matrix effect in each shellfish organ tissue, whole flesh or intravalvular liquid, corresponding matrix-matched standard sets were utilised (Section 4.1). Weighting 1/X was applied for calibration throughout the study. Subsequently, TTX concentrations in the samples were adjusted for recovery as assessed by the recovery experiments. In the absence of certified reference material for TTX analogues, these analogues were quantified against the TTX calibration curve, assuming an equimolar response. The ion ratio between the two MRMs was also used for confirmatory purposes. Results utilised for assessment of toxin profiles were absolute quantified values with no TEFs applied. Statistical analyses were performed using R software [68]. For all statistical tests, *p* values ≤ 0.05 were deemed statistically significant.

## 5. Conclusions

To our knowledge, this is the first report on TTX distribution in bivalve organs, collected systematically from their natural environment over time. Significantly higher concentrations of TTX in the oyster digestive system, compared to other organs, might indicate TTX accumulation through filter-feeding activities. Although the aim of our study was not to provide explanation about the source of TTX, the results provided in this paper are valuable and will enrich future discussion and research of TTX origin and accumulation in bivalves.

## Figures and Tables

**Table 1:** Extraction conditions and associated recovery of tetrodotoxin (TTX) in intravalvular fluid samples collected from *C. gigas*.

Treatment	Acidification	pH after Acidification	Heating (5 Min Boil)	TTX Recovery Mean ± s.d. and (RSD) ^1^
A	no	7.38	yes	57 ± 3 (6%)
B	no		no	91 ± 6 (7%)
C	Glacial acetic acid	3.75	yes	87 ± 3 (4%)
D	Glacial acetic acid		no	84 ± 5 (5%)
E	1% acetic acid	3.22	yes	66 ± 3 (5%)
F	1% acetic acid		no	68 ± 6 (8%)

^1^ TTX analysed by HILIC-MS/MS; values represent mean ± standard deviation (s.d.) and relative standard deviation (RSD).

**Table 2:** Effect on TTX detector signal and TTX recovery in different *C. gigas* matrices.

Matrix	r^2^	MM/Solvent Slope (%)	TTX Recovery Mean ± s.d. and (RSD) ^1^
Intravalvular fluid	0.997	104	91 ± 6 (7%)
Mantle	0.999	100	86 ± 8 (9%)
Gills	0.996	123	81 ± 6 (7%)
Whole flesh ^2^	0.995	117	76 ± 6 (8%)
Whole flesh ^3^	0.998	74	66 ± 2 (4%)
Adductor	0.999	83	68 ± 3 (5%)
Digestive gland	1.000	55	60 ± 5 (8%)
Palp	0.997	55	37 ± (12%)

^1^ Mean percentage recovery of TTX ± standard deviation (s.d.), spiked at a target concentration of 25 µg/kg in tissues and 50 ng/mL in intravalvular fluid, and the corresponding relative standard deviation (RSD); ^2^ TTX recovery in the whole flesh collected in March 2020; ^3^ TTX recovery in the whole flesh collected in May 2019.
